# Caractéristiques épidémio-cliniques du rétino blastome au Centre Hospitalier Universitaire Yalgado Ouedraogo du Burkina Faso: à propos de 32 cas

**DOI:** 10.11604/pamj.2020.37.269.20556

**Published:** 2020-11-25

**Authors:** Paté Sankara, Windinmanégdé Pierre Djiguimde, Ahgbatouhabéba Ahnoux-Zabsonre, Jérôme Sanou, Gertrude Meda-Hien, Ibrahim Abib Diomande, Mariam Dolo-Traore, Jean Wenceslas Diallo, Gabrielle Chantal Bouda, Gaoussou Sidibe

**Affiliations:** 1 Centre National de Lutte contre la Cécité, Ministère de la Santé, Ouagadougou, Burkina Faso,; 2 Service d´Ophtalmologie, Centre Hospitalier Universitaire de Bogodogo, Ouagadougou, Burkina Faso,; 3 Service d´Ophtalmologie, Centre Hospitalier Universitaire Yalgado Ouédraogo, Ouagadougou, Burkina Faso,; 4 Service d´Ophtalmologie, Centre Hospitalier Universitaire Bouaké, Université Alassane Ouattara, Bouaké, Côte d´Ivoire,; 5 Service d´Ophtalmologie, Centre Hospitalier Universitaire Sourô Sanou, Bobo-Dioulasso, Burkina Faso,; 6 Service de Pédiatrie, Centre Hospitalier Universitaire Yalgado Ouédraogo, Ouagadougou, Burkina Faso

**Keywords:** Rétinoblastome, Burkina Faso, cancer, enfant, chimiothérapie, chirurgie, Retinoblastoma, Burkina Faso, cancer, child, chemotherapy, surgery

## Abstract

Le rétinoblastome est la plus fréquente des tumeurs malignes oculaires de l´enfant. Son incidence est estimée à 1 cas pour 15 000 à 18 000 naissances. Nous nous sommes proposés de dégager ses aspects épidémio-cliniques et sa prise en charge au Burkina Faso. Etude rétrospective menée sur une période de 5 ans au Centre Hospitalier Universitaire Yalgado Ouédraogo. L´âge moyen de nos patients était de 33 mois avec une prédominance masculine (68,75%). L´exophtalmie avec 59,37% était le premier motif de consultation de nos patients qui présentaient en majorité une forme unilatérale (75%) à un stade d´extériorisation tumorale (59,38%). La chimiothérapie associée à la chirurgie était le traitement de choix avec une survie à 5 ans de 34,37%. Le rétinoblastome est un des cancers les plus fréquents chez les enfants de moins de 5 ans. La maladie est tardivement diagnostiquée dans nos pays à faibles revenus. La prise en charge est complexe avec un pronostic sombre en général. La mortalité et la morbidité dues à cette maladie sont démesurées dans nos pays aux plateaux techniques insuffisants. La prise en charge axée sur le dépistage précoce de la maladie et un traitement adapté doit être organisée dans nos pays à faibles revenus.

## Abstract (en)

Retinoblastoma is the most common intraocular cancer of childhood. Its estimated incidence is 1 case per 15 000 to 18 000 births. The purpose of this study is to highlight the epidemiological and clinical features of this disease as well as its management in Burkina Faso. We conducted a retrospective study over a period of 5 years at the Yalgado Ouedraogo University Hospital Center. The average age of patients was 33 months, with predominance of male sex (68.75%). Exophthalmia was the most frequent reason for consultation (59.37%). The predominant pattern was unilateral (75%) associated with eye protrusion (59.38%). Chemotherapy associated with surgery was the treatment of choice, with a 5-year survival rate of 34.37%. Retinoblastoma is one of the most common cancers in children younger than 5 years. In our low-income country this disease is diagnosed late. Patients´ management is complex and is commonly associated with poor prognosis. Mortality and morbidity from this disease are disproportionate in our country facing a shortage of technical equipment. In our low-income country, patient´s management should be based on early detection of the disease as well as on adequate treatment.

## Introduction

Le rétinoblastome est la plus fréquente des tumeurs malignes oculaires de l’enfant. Son incidence est estimée à 1 cas pour 15 000 à 18 000 naissances [1-3]. Le rétinoblastome fait partie des tumeurs malignes de l’enfant [4] autrefois considérées comme rares dans les pays d’Afrique. Ces tumeurs pédiatriques sont en pleine émergence du fait des possibilités diagnostiques mieux élaborées. Selon la littérature, les pathologies tumorales de l´enfant représenteraient 5,5% des causes de mortalité infantile. Elles constitueraient ainsi la quatrième cause de décès après les maladies infectieuses (40%), cardiovasculaires ou dégénératives (19%) et la mortalité périnatale (8%) [5,6]. Dans l´optique de faire le point de cette pathologie en dégageant ses aspects épidémio-cliniques et sa prise en charge au Burkina Faso, nous nous sommes proposés de mener cette étude.

## Méthodes

Il s´est agi d´une étude transversale à visée descriptive qui s´est déroulée sur une période de 5 ans allant du 1^er^janvier 2013 au 31 décembre 2017 dans les services d´ophtalmologie et d´oncologie pédiatrique du Centre Hospitalier Universitaire, Yalgado Ouédraogo (CHU-YO). Elle était basée sur la collecte de données des dossiers des enfants suivis pour rétinoblastome. Ont été inclus dans notre étude les patients âgés de 0 à 15 ans suivis à la fois dans les services d´Ophtalmologie et d´Oncologie Pédiatrique du CHU-YO pour rétinoblastome. N´ont pas été inclus à cette étude les patients présentant un rétinoblastome mais n´étant pas suivis concomitamment dans les 2 services précités. Une fiche de collecte individuelle a été établie à cet effet renseignant sur les aspects épidémiologiques (âge, sexe, niveau d´instruction…), cliniques (signes fonctionnels, examen à la lampe fente, et fond d´œil sous anesthésie générale) et sur la prise en charge thérapeutique (chimiothérapie, chirurgie). La Classification Internationale du Rétinoblastome (ICRB) a été utilisée pour déterminer le stade clinique des tumeurs. La chimiothérapie était associée ou non à la chirurgie selon le stade clinique après réalisation d´un bilan sanguin, cardiaque, rénal, hépatique, et nutritionnel. Le traitement chimique d´attaque préopératoire et postopératoire d´entretien a été réalisé selon le protocole du Groupe Franco-Africain d´Oncologie Pédiatrique (G.F.A.O.P) utilisant les produits suivants à des doses et des jours (J) précis: Etoposide (VP16) (150mg/m^2^/jr) de J1 a J3, Carboplatine (200mg/m^2^/jr) de J1 a J3,Vincristine (1,5mg/m^2^/jr) à J1 et Cyclophosphamide (300mg/m^2^/jr) de J1 a J5. Les données ont été saisies et traitées à l´aide du logiciel Epi info 7.1.3. Les tableaux et graphiques ont été réalisés à l´aide du logiciel EXCEL 2013.

## Résultats

**Figure 1:**
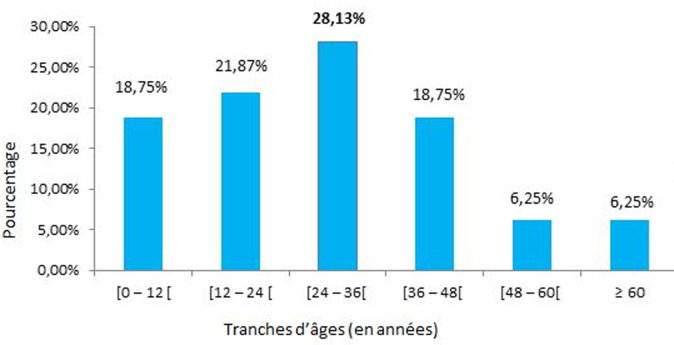
répartition des patients selon l´âge

**Figure 2:**
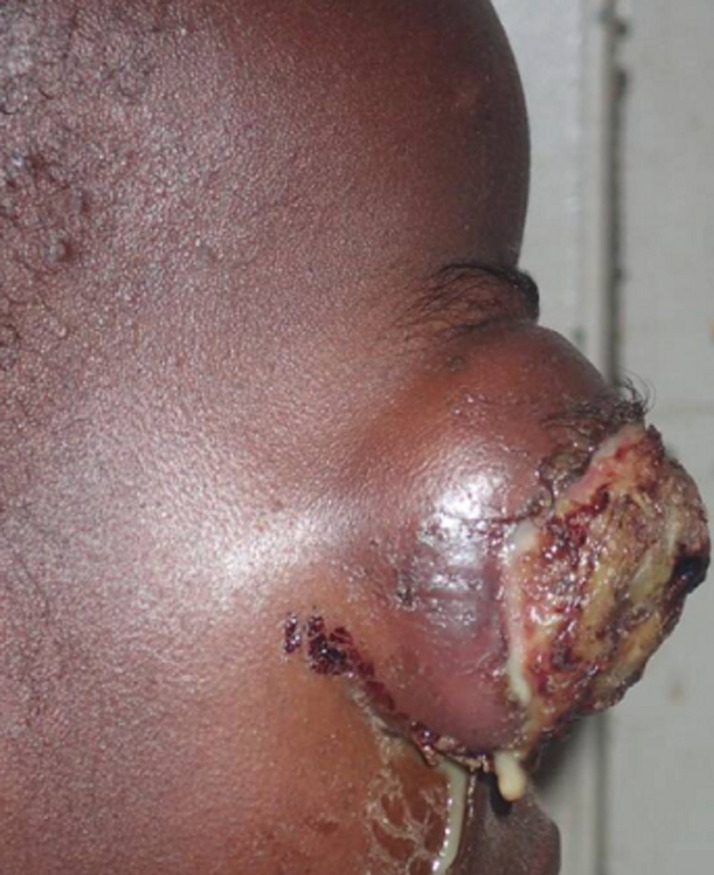
exophtalmie montrant l´aspect bourgeonnant d´un rétinoblastome

**Figure 3:**
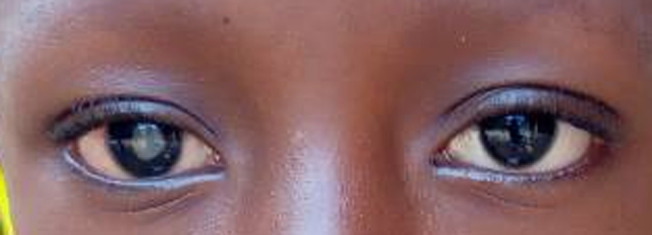
aspect d´une leucocorie à l´œil droit

**Figure 4:**
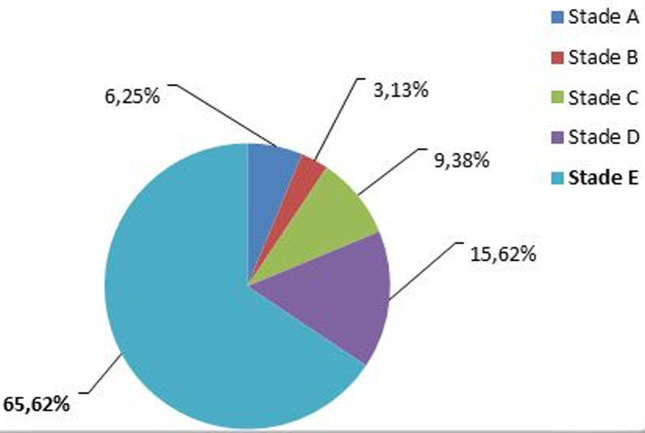
répartition des patients selon la classification A

Sur un total de 10142 enfants ayant consulté pendant la période d´étude, il a été répertorié 106 cas de rétinoblastomes soit une fréquence de 1,04%. Notre étude a été menée pour autant sur 32 de ces cas qui répondaient à nos critères d´inclusions soit une fréquence relative de 30,19% de l´ensemble des rétinoblastomes. L´âge moyen de nos patients était de 33 mois avec des extrêmes de 1 mois et de 96 mois (Figure 1). La majorité de nos patients était de sexe masculin (68,75%), correspondant à un sex-ratio de 2,2. L´exophtalmie (Figure 2) avec 59,37% était le motif de consultation le plus fréquent suivie de la leucocorie (Figure 3) avec (31,25%). La majorité des patients (53,12%) a consulté dans un délai de 3 à 180 jours avec un délai moyen de 206 jours. Les formes unilatérales (75%) avec prédominance du côté droit (53,13%) représentaient la majorité des cas. La tumeur était à un stade d´extériorisation dans la majorité des cas (59,38%) chez nos patients (Tableau 1). L´imagerie montrait en majorité (65,62%) une masse tumorale envahissante occupant la presque totalité de la cavité orbitaire (Tableau 2). Le stade E avec 65,62% prédominait le tableau clinique selon la Classification Internationale du Rétinoblastome (ICRB) (Figure 4). L´association chimiothérapie-chirurgie était le protocole thérapeutique de premier choix pour nos patients avec la réalisation d´une exentération totale dans 59,37% des cas. La survie à 5 ans était de 34,37%.

**Tableau 1:** répartition des patients selon les aspects cliniques

Aspects cliniques	Effectif	Pourcentage (%)
Leucocorie avec petite tumeur visible au FO	2	6,25
Leucocorie avec tumeur visible au FO et DR	4	12,50
Tumeur au FO occupant tout le pôle postérieur, DR et essaimage intra vitréen localisé ou diffus	7	21,87
**Extériorisation tumorale**	**19**	**59,38**
Total	32	100

**Tableau 2:** répartition des patients selon l´imagerie

Aspects cliniques	Effectif	Pourcentage (%)
Masse tumorale intraoculaire bien limitée avec calcification	3	9,38
Masse tumorale intraoculaire irrégulière avec calcification, dissémination intra vitréenne et décollement rétinien	8	25
Masse tumorale avec calcification envahissant tout le GO et/ou la cavité orbitaire	21	65,62
Total	32	100

## Discussion

A l´issue de cette étude, nous avons noté que le rétinoblastome avait une prévalence hospitalière de 1,04% dans le Service de Pédiatrie du CHU-YO. Ce résultat confirme ainsi qu´il s´agit d´une tumeur rare mais qui demeure pour autant la tumeur oculaire de l´enfant la plus fréquente. Il s´agit d´une tumeur hautement maligne dont le diagnostic le plus souvent tardif dans nos milieux est responsable d´un taux de mortalité encore très élevé. L´âge moyen de nos patients était de 33 mois (2 ans et 9 mois) avec des extrêmes de 1 mois et de 8 ans. Nos résultats sont superposables à ceux d´Hassan [7] en Egypte qui notaient dans leurs études respectives un âge moyen de 2 ans 8 mois et de 3 ans. Ces différents âges moyens témoignent du fait que le rétinoblastome surviendrait préférentiellement chez le jeune nourrisson avec une prédominance masculine constatée dans notre étude qui trouvait une sex-ratio de 2,2. Cependant, une grande étude multicentrique réalisée par Moll *et al*. [1] recherchant une corrélation entre le rétinoblastome et le genre n´a pas trouvé de différence significative. Elle confirme ainsi que le rétinoblastome surviendrait aussi bien chez les sujets de sexe masculin que chez les sujets sexe féminin. L´exophtalmie avec 59,37% a été le premier motif de consultation de nos patients suivie de la leucocorie (31,25%). Owoeye *et al*. [8] au Nigéria avait aussi noté 84,6 % d´exophtalmies dans son étude clinique réalisée en 2006. Ces constats révèlent le caractère tardif de consultation des patients dans nos milieux africains.

En effet, différents paramètres pourraient expliquer cette réalité. Les pratiques ancestrales (tradithérapie, fétichisme, valeurs culturelles…) sont le plus souvent utilisées en première intention pour le traitement de nombreuses pathologies [9,10]. L´inefficacité de ces pratiques constatée après une longue période associée à une exophtalmie d´installation progressive chez un sujet jeune, imposent la consultation dans un centre de santé pour la prise en charge. Par ailleurs la pauvreté de la population associée à la rareté des centres de santé avec un plateau technique insuffisant sont aussi des facteurs pouvant expliquer le retard de consultation des patients [10-12]. Les formes unilatérales du rétinoblastome ont été les plus fréquentes dans notre étude (75%) ce qui est corroboré dans la littérature par Diomande *et al*. [12] aux Etats-Unis en 1995 après une étude multicentrique réalisée sur le rétinoblastome. Le même constat est fait par Jaradat *et al*. [13] en Jordanie en 2011 à l´issue d´une étude épidémiologique réalisée sur le rétinoblastome. L´unilatéralité prédominante du rétinoblastome a une signification génétique s´expliquant par le fait que l´expression clinique du rétinoblastome nécessite au préalable une mutation de deux allèles du gène correspondant. Elle peut se faire simultanément dans la cellule rétinienne au cours du développement fœtal responsable des formes unilatérales, ou alors de façon dissociée avec une première mutation génétique constitutionnelle présente dans toutes les cellules de l´organisme du patient suivi de la seconde mutation au niveau de la cellule rétinienne qui sera responsable du rétinoblastome bilatéral. La rareté de cette forme de mutation dissociée explique la faible fréquence des formes dites bilatérales. La leucocorie qui est un signe clinique principal d´appel dans les formes débutantes du rétinoblastome n´a été observée que chez certains de nos patients (31,25%). A contrario sa fréquence est assez élevée dans les pays développés comme constaté dans l´étude de Chawla *et al*. [14] réalisée en Inde en 2016. Ce qui se conçoit aisément du fait d´un diagnostic précoce dans ces pays facilité par une plus grande accessibilité aux structures sanitaires disposant d´un plateau technique adapté.

Les formes extériorisées avec protrusion du globe dominaient notre tableau clinique. En effet, elles ont été observées chez 59,38% de nos patients. Nos résultats sont superposables aux 84,6 % d´exophtalmies retrouvées par Owoeye *et al*. [8] au Nigéria. Ces tableaux cliniques sont le reflet du diagnostic tardif de cette pathologie dans nos milieux africains. Ces exophtalmies témoignent de la propagation des cellules tumorales malignes au-delà des limites oculo-orbitaires rendant complexe la prise en charge, et le plus souvent témoin d´un pronostic sombre. Ces exophtalmies sont le plus souvent associées à des lésions des structures antérieures et postérieures du globe oculaire se traduisant parfois par des nécroses cornéennes, un pseudo-hypopion en chambre antérieure, un essaimage vitréen de particules tumorales le tout évoluant parfois dans un contexte infectieux, comme observées chez certains de nos patients. Ce tableau clinique ainsi décrit permet de comprendre la prédominance du stade E (65,62%) selon la Classification Internationale du Rétinoblastome (ICRB). Cette prédominance du stade E a aussi été relevée par Parrilla-Vallejo *et al*. [15] dans une étude clinique récente réalisée en Espagne sur le rétinoblastome. Ces aspects ainsi décrits se traduisaient en imagerie par la présence de masses tumorales envahissantes avec des calcifications pathognomoniques du rétinoblastome [16]. Concernant la prise en charge thérapeutique des patients, le protocole chimiothérapie première associée à la chirurgie a été le plus pratiqué (53,13%). Il avait pour but de réduire le volume tumoral qui était secondairement réséquée chirurgicalement par exentération totale. Une chimiothérapie secondaire pour détruire d´éventuelles cellules tumorales résiduelles était prévue chez nos patients mais n´a pu être réalisée pour diverses raisons (perdus de vue, pauvreté, décès). La chimiothérapie associée à la chirurgie a aussi été le protocole pratiqué par Owoeye *et al*. [8] au Nigéria en 2006. La survie à 5 ans de nos patients demeure sombre quels que soient les protocoles qui pourraient être adaptés à ceux-ci, vu le stade évolué de la maladie en général au moment du diagnostic.

## Conclusion

Le rétinoblastome est un des cancers les plus fréquents chez les enfants de moins de 5 ans. La mortalité et la morbidité dues à cette maladie sont démesurées dans nos pays à faibles revenus associés à l´insuffisance des plateaux techniques. L´organisation d´une prise en charge axée sur le dépistage précoce de la maladie et un traitement adapté permettrait la réduction de ses complications et l´amélioration de son pronostic vital dans nos pays à faibles revenus.

### Etat des connaissances sur le sujet

- Le rétinoblastome est une tumeur oculaire hautement maligne;
- Sa prise en charge est assez bien codifiée avec guérison et conservation oculaire dans la plupart des cas dans les pays développés;
- Les formes avancées sont toujours observées dans les pays sous-développés.

### Contribution de notre étude à la connaissance

- Le diagnostic du rétinoblastome demeure tardif dans les pays en voie de développement et particulièrement au Burkina Faso;
- L´exophtalmie est malheureusement la circonstance de découverte la plus fréquente au Burkina Faso;
- Il y a une impérieuse nécessité d´améliorer les plateaux techniques pour un diagnostic précoce et une prise en charge adaptée.
